# Supplementary material for: Full-cycle device-scale simulations of memory materials with a tailored atomic-cluster-expansion potential
Source: Nat Commun. 2025 Sep 30;16:8688. doi: 10.1038/s41467-025-63732-4 (PMC12484596; doi:10.1038/s41467-025-63732-4)
Supplement: Supplementary file 1 — Supplementary Information [file 41467_2025_63732_MOESM1_ESM.pdf]

Supplementary Information for

Full-cycle device-scale simulations of memory materials with a tailored atomic-cluster-expansion potential

Yuxing Zhou,[1] Daniel F. Thomas du Toit,[1] Stephen R. Elliott,[2] Wei Zhang,[3,*] and Volker L. Deringer[1,*]

[1]*Inorganic Chemistry Laboratory, Department of Chemistry, University of Oxford, Oxford OX1 3QR, United Kingdom*

[2]*Physical and Theoretical Chemistry Laboratory, Department of Chemistry, University of Oxford, Oxford OX1 3QZ, United Kingdom*

[3]*Center for Alloy Innovation and Design (CAID), State Key Laboratory for Mechanical Behavior of Materials, Xi'an Jiaotong University, Xi'an 710049, China*

*E-mail: wzhang0@mail.xjtu.edu.cn; volker.deringer@chem.ox.ac.uk

This document contains:

Supplementary Note 1
Components of the reference dataset S2

Supplementary Note 2
Validation and computational efficiency tests S3

Supplementary Note 3
Simulation protocols for device-scale modelling S4

Supplementary Tables S5–S6
Supplementary Figures S7–S13
Supplementary References S14

Supplementary Note 1 (Components of the reference dataset)

We here provide more details about the construction of the reference dataset for the ACE potential model fitted in the present work (referred to as "GST-ACE-24" in the following). An overview of the fitting protocol for GST-ACE-24 is shown in Fig. 1b of the main text. We started from the training dataset of our previously published GAP potential[S1] (referred to as "GST-GAP-22" in the following). We first re-labelled the GST-GAP-22 dataset using DFT with the Perdew–Burke–Ernzerhof (PBE) exchange–correlation functional[S2]. We added 59 amorphous and supercooled liquid structures of different GST compositions into the iter-0 dataset: these AIMD configurations were taken from melt-quench AIMD simulations using PBE, reported in Ref. [S1], and re-labelled here using single-point DFT computations. We then carried out the first XPOT-based hyperparameter optimisation.

Next, five iterations were carried out to gradually expand the training dataset for the ACE potential. From iter-1 to iter-3, we added domain-relevant configurations, including the melt-quenched structures and the intermediate configurations during the crystallisation and melting processes. In these iterations, the previous version of the ACE model was used to run independent ACE-driven MD (ACE-MD) simulations, in which new structural models were collected randomly from the ACE-MD trajectories. To obtain the domain-specific configurations for crystallisation, we started from crystal structures of GST. Some atomic layers were manually fixed during the melt-quench simulations. We then annealed these partially crystalline structures at elevated temperatures (e.g., 500–700 K). Due to the existence of pre-fixed crystalline layers (or seeds), the structural models started to recrystallise. We then randomly took intermediate configurations during the recrystallisation processes and added them into the training dataset. To collect the domain-specific configurations for melting, we started from crystal structures of GST. We then heated the crystal structures from 300 K to above their melting points. During the heating process, we collected the configurations in which some local structural disordering started to appear, which represented the starting of melting.

In addition, to increase the robustness of the ACE model, we added some small-scale (6–40 atoms) hard-sphere random structures of different chemical compositions in iter-1 to iter-3, generated using the `buildcell` code of the *ab initio* random structure searching (AIRSS) code[S3,S4]. As shown in the ablation studies presented in the main text (cf. Fig. 2), including some random structures with short atomic distances can help prevent failures caused by lost atoms in ACE-MD simulations. After iter-3, we performed a second XPOT run (which, however, did not notably improve the model, and therefore we continued with the previous hyperparameters).

To further improve the robustness of our ACE models in ACE-MD simulations, we also carried out ACE-driven random structure searching (ACE-RSS, akin to AIRSS[S3,S4] and GAP-RSS[S5,S6]) in two other iterations (iter-4 and iter-5). We note that ACE-RSS could not be carried out in iter-1 to iter-3, because some relaxations failed, due to lost atoms. However, as more randomly generated structures were accumulated, successful ACE-RSS runs further explored the potential-energy surface by incorporating the relaxation of such random structures.

The resultant training dataset included 1,944 new training structures. All structures were labelled with the PBE functional. A summary of the composition of the training dataset for GST-ACE-24 is given in Supplementary Table 1.

Supplementary Note 2 (Validation and computational efficiency tests)

We carried out comprehensive validation studies of our ACE models, including numerical and physically-guided validation, following ideas discussed in Ref. [S7]. We performed comprehensive ablation studies, as shown in Fig. 2 of the main text. In these ablation studies, we gradually removed the training structures from the GST-ACE-24 dataset, or we changed the hyperparameters used in the ACE models. We constructed a testing dataset which consists of conventional disordered GST structures (containing ≈ 200 atoms) and intermediate configurations during the phase-transition processes (containing 1,008 atoms). The former were taken from melt-quench AIMD simulations, as reported in Ref. [S1], and the latter were taken from AIMD crystallisation simulations, as shown in Ref. [S8]. This testing dataset was labelled using DFT with PBE, and we then computed the root mean square error (RMSE) of energy and forces for different ACE models, based on the testing dataset.

To test the computational efficiency of GST-ACE-24, as compared to the previously reported GST-GAP-22 model, we carried out ACE- and GAP-driven MD simulations on the CPU nodes of ARCHER2, a high-performance computing system in the UK. The compute nodes each have 128 cores, which are dual-socket nodes with two 64-core AMD EPYC 7742 processors (see details at Ref. [S9]). The same MD protocol was used to evaluate the computational efficiency: the structural models were annealed at 1,000 K for 2 ps (i.e., 1,000 MD steps). The total running time was measured to indicate the computational speed. The memory per node is 256 GB (standard nodes) or 512 GB (high-memory nodes). A large efficiency gap was observed between ACE-MD and GAP-MD—ACE-MD is more than 400 times faster than GAP-MD. More importantly, GAP-MD simulations require larger memory sizes, and simulations of more than 1 million atoms failed due to the lack of memory. In contrast, ACE-MD is more memory-efficient and can be used in device-scale simulations up to one billion atoms (cf. Fig. 1 in the main text and Supplementary Fig. 1).

Supplementary Note 3 (Simulation protocols for device-scale modelling)

We here discuss the construction of structural models of different device setups[S1] and the simulation protocols for the device-scale modelling performed in Ref. [S1] and in the present work.

We first discuss how to construct the structural models of $Ge_1Sb_2Te_4$ for two different device architectures, *viz.* the cross-point and mushroom-type devices. For the cross-point device, we used a simulation box of 20 × 20 × 40 nm^3 which contains 532,980 atoms, as in Ref. [S1]. We first constructed an amorphous slab of $Ge_1Sb_2Te_4$ (20 × 20 × 0.8 nm^3) via a melt-quench simulation. This slab was placed on the top of the cell and was fixed during all ACE-MD simulations, serving as a heat barrier to stop unwanted atomic migrations across the periodic box. To construct a structural model for mushroom-type devices, we built a 100 × 40 × 5 nm^3 slab model (containing 794,808 atoms), which represents the cross-section of 100 × 40 nm^2 in the middle of a mushroom-type cell (cf. Fig. 4b in the main text). We also fixed a 6-nm thick slab of amorphous $Ge_1Sb_2Te_4$ on the top of this cross-section model as a heat barrier. We note that these heat barriers shown in both structural models reflect the thermal barriers that are in contact with the GST material in real-world GST devices.

Next, we describe how to simulate the non-isothermal heating and cooling processes caused by the RESET pulses. To simulate the non-isothermal heating process, which is triggered by a short and intense external heating pulse, we used the NVE ensemble. We gradually added kinetic energy to the atoms in the programming regions. These added kinetic-energy values were spatially inhomogeneous, in a linear way along the *z*-axis. More energy was added to the atoms close to the bottom, whereas less energy was added to the atoms on the top. By doing this, a temperature gradient from the bottom to the top of the cell was created (Supplementary Figures 2 and 3), reflecting a non-isothermal heating process.

To model the heat-dissipation process after the heating pulse, we gradually removed the kinetic energy from the atoms in the programming region. The amount of energy removed from a given atom depends on its atomic temperature. We note that the "atomic temperature" discussed here is not a direct physical observable, but a concept used in MD simulations to describe the kinetic energy associated with the motion of an individual atom. In other words, the atomic temperature is instantaneous and is defined directly, based on its instantaneous kinetic energy (and velocity),

$$T_{\mathrm{atom}} = \frac{2}{3k_{\mathrm{B}}} \cdot E_{\mathrm{kin}} = \frac{2}{3k_{\mathrm{B}}} \cdot \frac{1}{2} m v^2, \tag{S1}$$

in which T_{atom} is the instantaneous atomic temperature, E_{kin} is the instantaneous kinetic energy, k_{B} is the Boltzmann constant, m is the atomic mass, and v is the instantaneous velocity obtained from MD simulations. The instantaneous atomic temperature of a given atom will be compared to ambient temperature (i.e., 300 K). The higher the instantaneous atomic temperature, the greater the amount of kinetic energy that will be removed. By doing this, we simulated the heat-dissipation process, which also showed prominent temperature gradients (Supplementary Figures 2 and 3).

In contrast, the SET (crystallisation) processes in the present work were all modelled using the NVT ensemble. A Langevin thermostat, with a damping timescale of 0.04 ps (corresponding to a damping coefficient of 25 ps^{-1}), was used to control the temperature.

Supplementary Tables

Supplementary Table 1. Summary of the composition of the GST-ACE-24 reference dataset.

Components	Descriptions	database size	
		**Cells**	**Atoms**
GST-GAP-22 (published in Ref. [S1])	Free atom	3	3
	Dimer	210	420
	Crystalline structures	1261	69055
	AIMD structures (PBEsol level)	210	41490
	Melt-quenched disordered structures	336	65962
	Phase-transition configurations	672	164202
iter-0	AIMD structures (PBE level)	59	11484
iter-1	Melt-quenched disordered structures	196	30208
	Phase-transition configurations	168	43752
	Hard-sphere random structures	120	2317
iter-2	Melt-quenched disordered structures	140	25560
	Phase-transition configurations	167	43493
	Hard-sphere random structures	55	1390
iter-3	Melt-quenched disordered structures	140	25560
	Phase-transition configurations	168	43752
	Hard-sphere random structures	144	2090
iter-4	ACE-RSS structures	287	4602
iter-5	ACE-RSS structures	300	4132
**Total**		**4636**	**579472**

Supplementary Table 2. Optimised hyperparameters in the XPOT runs.

Optimised hyperparameters	Descriptions	Hyperparameter ranges	Values after optimisation
Cut-off	Cut-off distance (Å)	5.5 – 8.0	8.0
Radial parameter	Used in the construction of radial basis	4 – 10	10
Prefactor k_2	Used to define the embedding functions: $E_i = \varphi + k_2\sqrt{\varphi} + k_3\varphi^2$	0.1 – 5	4.577
Prefactor k_3		0.1 – 5	0.101

Supplementary Figures

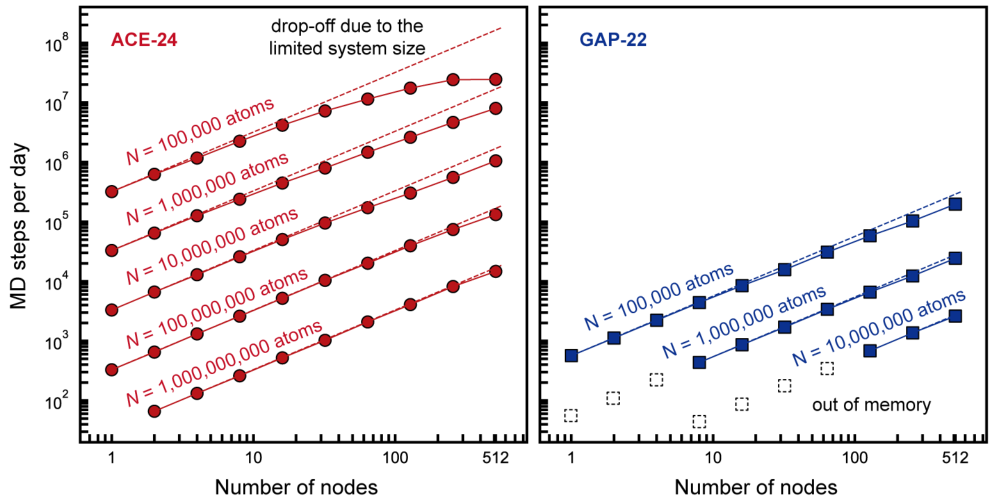

Supplementary Fig. 1: Strong scaling tests for GST-ACE-24 and GST-GAP-22. To evaluate the computational speed, a 2-ps (i.e., 1,000 MD steps) molecular-dynamics (MD) simulation was carried out. The numbers of MD steps achieved per day were then calculated and are plotted against the number of nodes. Different system sizes were tested, ranging from 100,000 atoms to 1,000,000,000 (1 billion) atoms; the results of GST-ACE-24 and GST-GAP-22 are indicated by filled circles and squares, respectively. The dashed lines indicate ideal strong scaling behaviour. Note that some data points could not be measured when using GST-GAP-22 in the simulation of 1,000,000 atoms or more ("missing" filled squares in the right-hand panel), because GAP-MD failed due to a lack of memory: in these cases, we show empty dashed squares extrapolated from the ideal scaling lines.

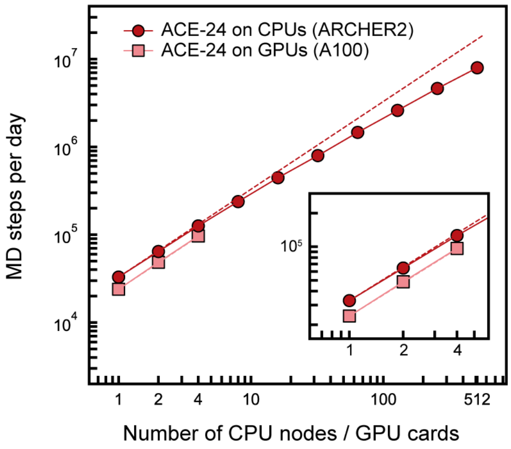

Supplementary Fig. 2: Performance comparison of ACE-MD simulations for a 1-million-atom system on CPU and GPU resources. ACE-MD simulations using CPU were performed on ARCHER2, in which each node has 128 cores. GPU-based benchmarks were performed on up to 4 interconnected NVIDIA A100 cards on a single server. The dashed lines indicate ideal scaling behaviour for CPU and GPU, respectively.

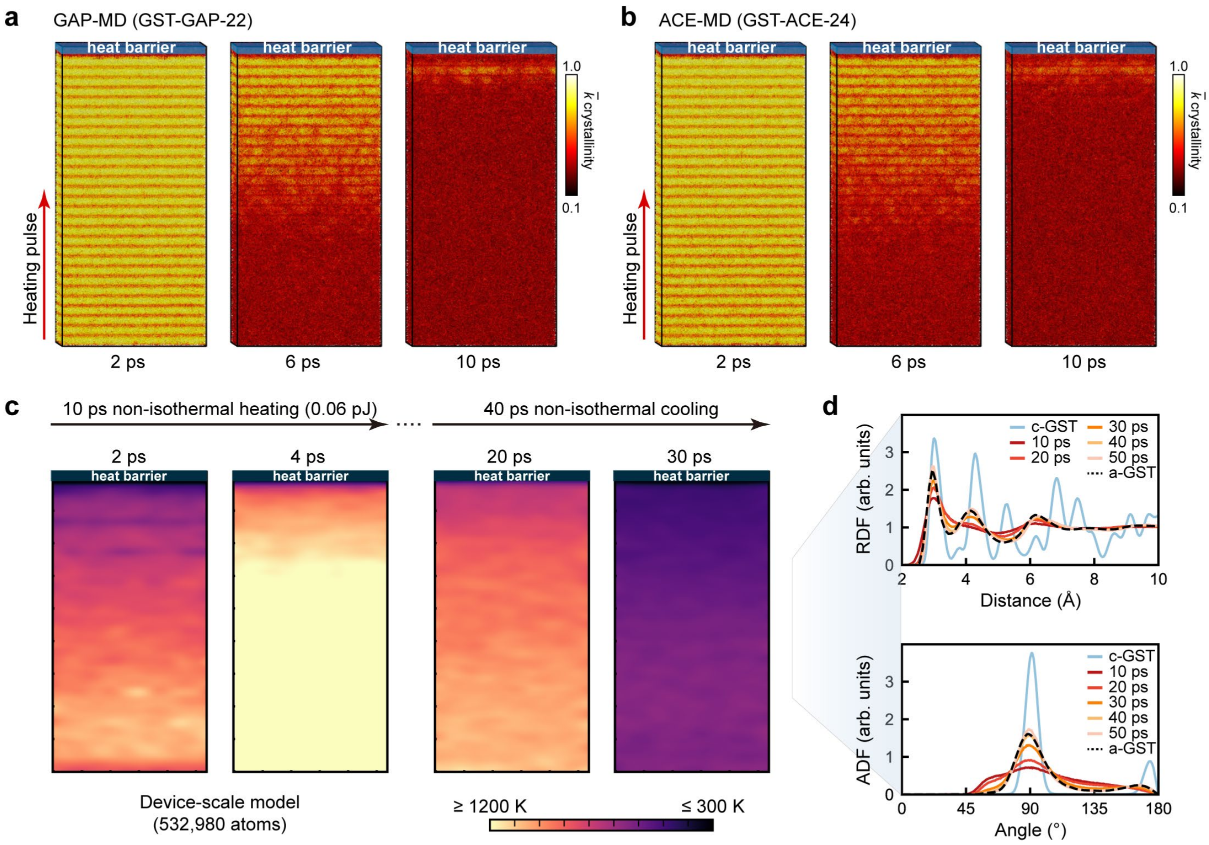

Supplementary Fig. 3: The non-isothermal RESET process of GST-based cross-point devices. (**a**) Snapshots of a device-scale GAP-MD simulation (532,000 atoms) for $Ge_1Sb_2Te_4$, which was performed in this work using the GST-GAP-22 potential[S1]. The initial configuration is the (single-crystal) trigonal phase of $Ge_1Sb_2Te_4$. An ≈ 8 Å thick slab of amorphous GST was fixed on the top of the cell (near z = 40 nm), serving as a heat barrier to stop atoms from migrating across the boundary of the periodic box. This setup differs from the initial structural model in Ref. [S1], where a crystalline GST slab was used as the heat barrier. A 10-ps heating pulse (0.064 pJ) was added from the bottom to the top of cell to melt the structural model. (**b**) The same type of heating simulation as shown in panel (a), but now carried out using our new GST-ACE-24 potential. Colour coding in panels (a) and (b) indicates the SOAP-based per-atom crystallinity[S8], $\bar{k}$, suggesting a gradual melting process from crystalline-like (yellow) to amorphous-like (red). (**c**) A simulation of the 40-ps heat-dissipation process was carried out using ACE-MD after the 10-ps heating pulse. The corresponding two-dimensional temperature profiles were calculated from the ACE-MD simulations for the non-isothermal RESET process, based on the approach described in Ref. [S1]. (**d**) The evolution of the GST structure from this heating-and-cooling process, characterised using both RDF and ADF results from the central area of the device-scale model (20 nm thick). We note that the structural features of amorphous $Ge_1Sb_2Te_4$ obtained from the device-scale structural model agree well with those of small-scale bulk amorphous structures, modelled by AIMD in our previous work (Ref. [S1]). This figure is drawn in a style similar to Ref. [S1].

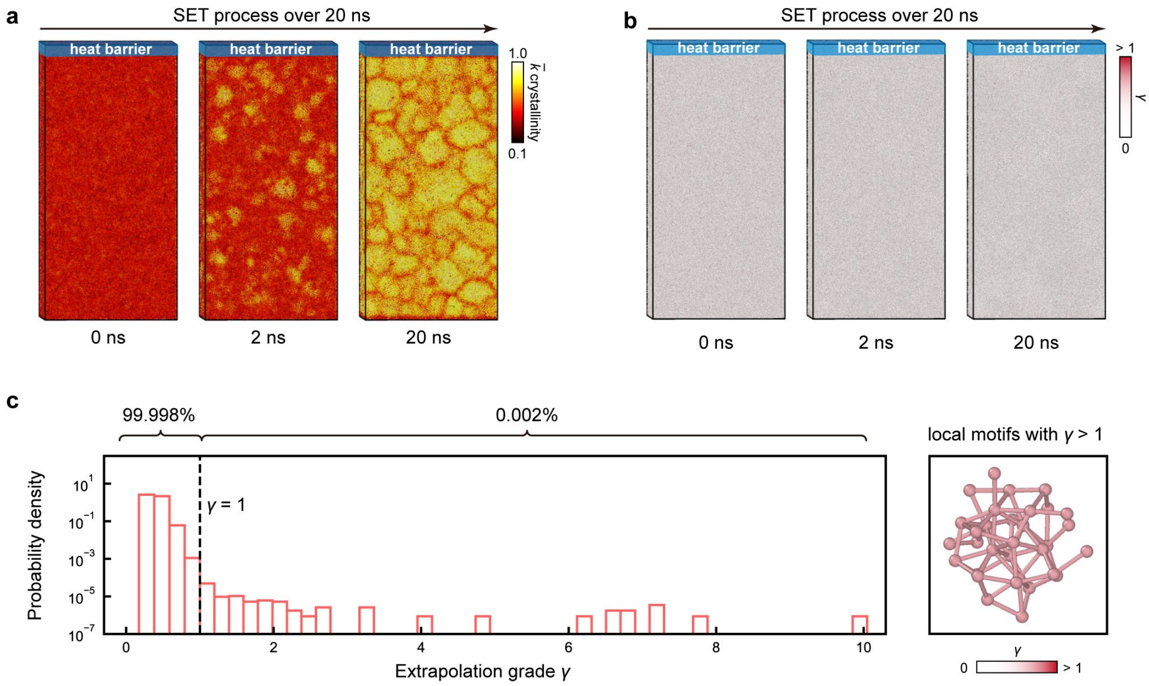

Supplementary Fig. 4: The structural evolution during the 20-ns SET process simulation, quantified using: (**a**) SOAP-based per-atom crystallinity[S8], $\bar{k}$, and (**b**) per-atom uncertainty of the ACE prediction, indicated by the extrapolation grade, γ (see Ref. [S10]). Higher γ values indicate greater model uncertainty about the corresponding local atomic motifs. (**c**) Histogram of the per-atom γ values of all atoms during the 20-ns SET process, drawn in a style similar to Ref. [S11]. A threshold value of $\gamma = 1$ (dashed line) separates interpolative ($\gamma \leq 1$) from extrapolative ($\gamma > 1$) regimes[S10]. An example of a highly uncertain local motif is shown on the right, where all atomic γ values are greater than 1.

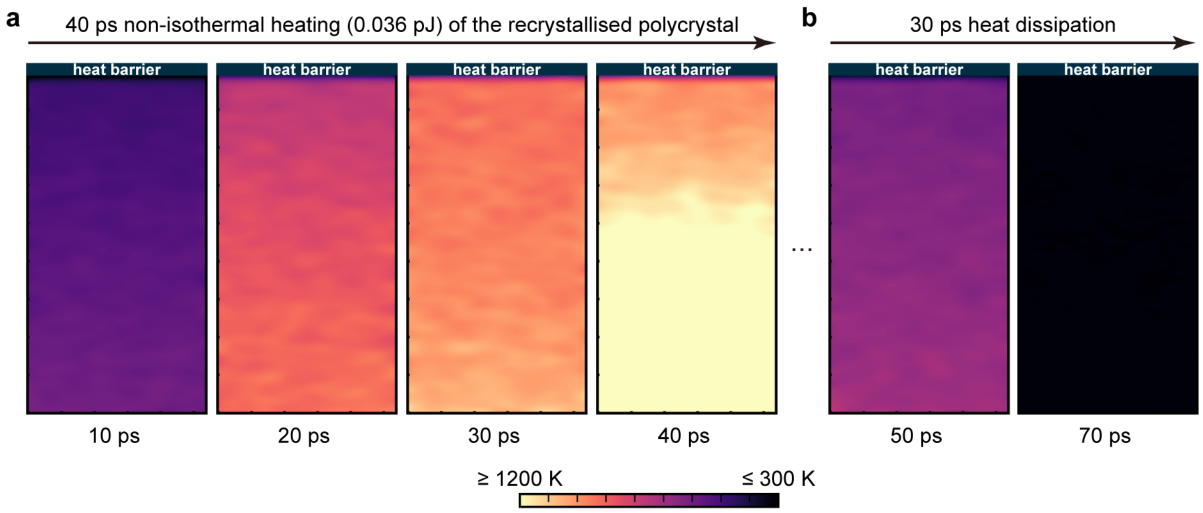

Supplementary Fig. 5: Temperature profiles of the RESET process for the recrystallised polycrystalline structure. The RESET process included: (**a**) a 40-ps heating (0.036 pJ) process; and (**b**) a 30-ps heat dissipation process. The initial configuration of this RESET simulation was taken from the recrystallised polycrystalline structure of $Ge_1Sb_2Te_4$ (cf. Fig. 3c in the main text). The atomic trajectory of this RESET process is shown in Fig. 3c of the main text. The technical details regarding the computation of the temperature profiles have been given in our previous work (Ref. [S1]).

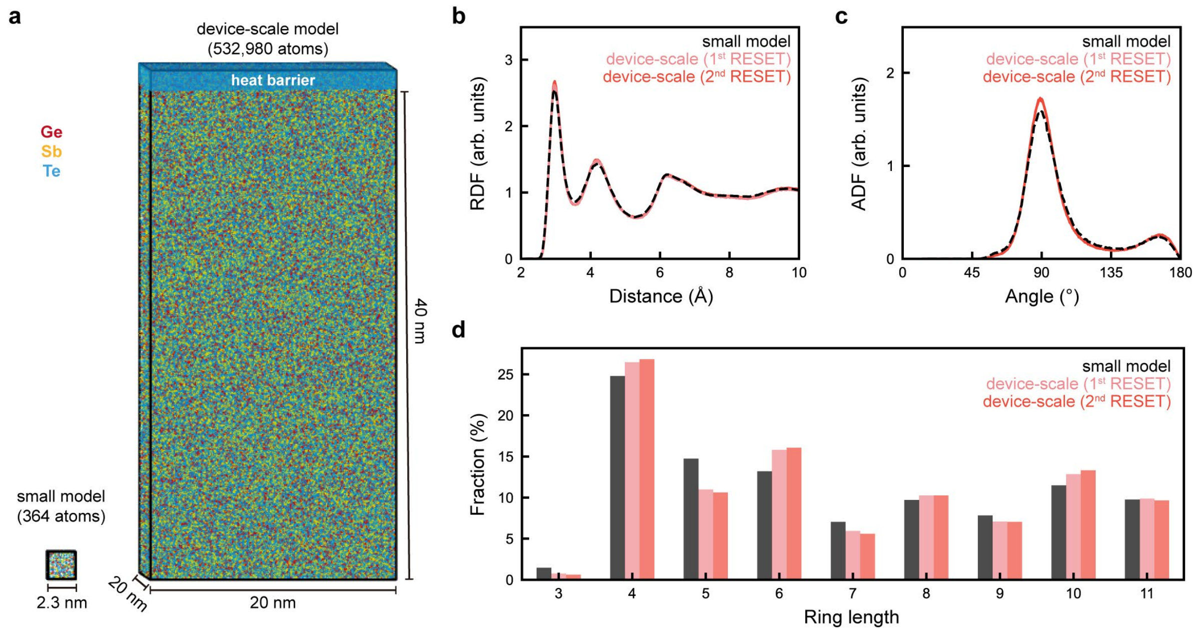

Supplementary Fig. 6: Structural analyses of amorphous GST models with different sizes. (**a**) Snapshots of amorphous GST, including a small-scale model (364 atoms) that was generated via a melt-quench process using ACE-MD, and a device-scale model (532,980 atoms). Ge, Sb, and Te atoms are rendered as red, yellow, and blue, respectively. (**b**) Computed radial distribution function (RDF) values, including analysis for device-scale structural models obtained after the first and second RESET pulse, respectively (cf. Fig. 3 in the main text). Only the central volumes of the device-scale models ($20 \times 20 \times 20$ nm^3) were used for structural analyses. (**c**) Same but for computed angular distribution function (ADF) values. (**d**) Same but for the distribution of ring sizes.

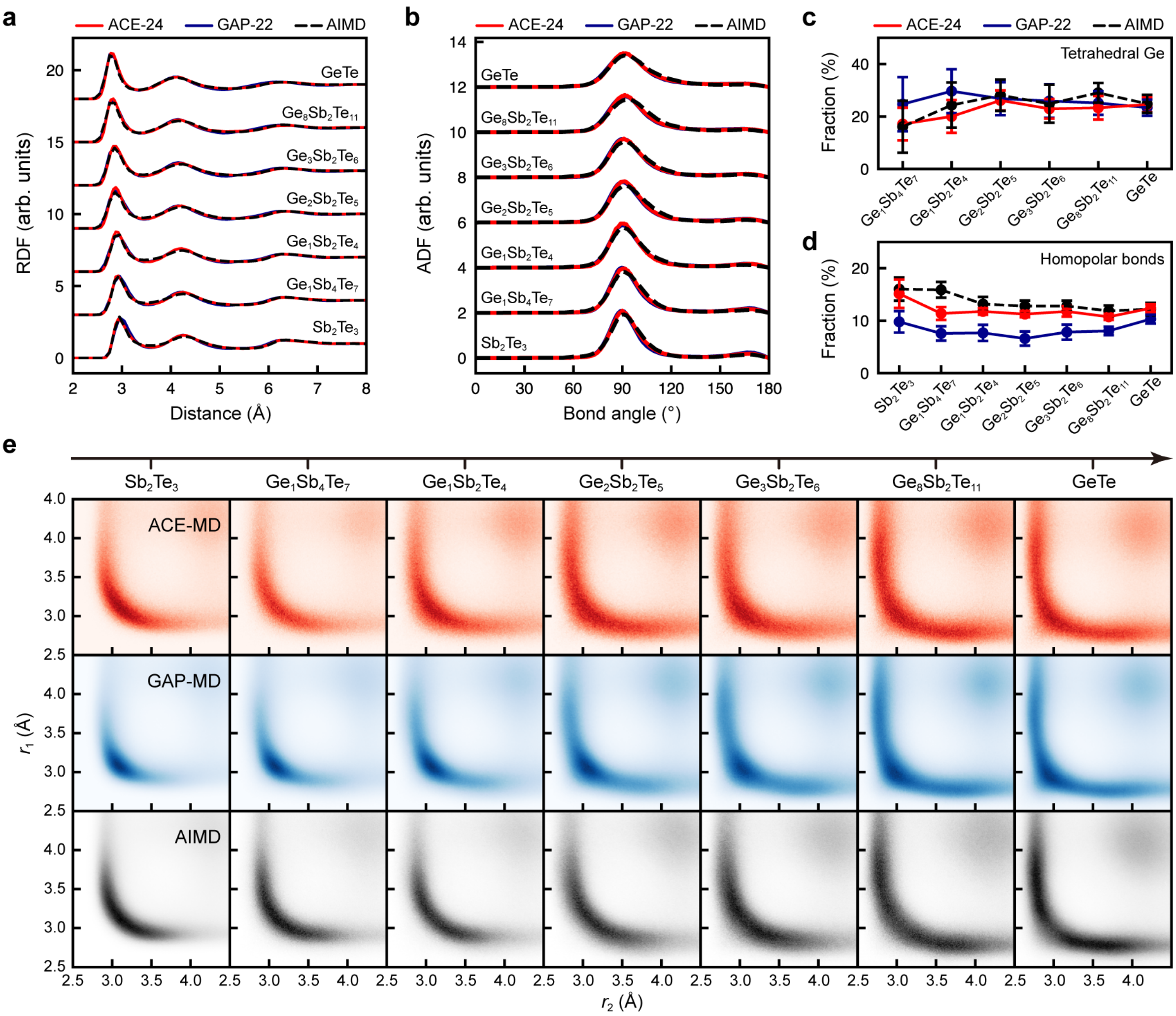

Supplementary Fig. 7: Local structure of amorphous GST compositions from ML-driven MD and AIMD simulations. (**a**) Radial distribution functions (RDFs) for seven different compositions along the GeTe–Sb_2Te_3 tie-line. (**b**) Angular distribution function (ADF) for the seven compounds. (**c**) The calculated fraction of tetrahedral Ge atoms, defined by a bond-order parameter, as discussed in previous work[S12]. (**d**) Fraction of homopolar and "wrong" bonds, i.e., bonds between two cation-like atoms or two anion-like atoms (viz. Ge–Ge, Ge–Sb, Sb–Sb, and Te–Te). (**e**) Angular-limited three-body correlation (ALTBC) functions for the amorphous structures of seven GST alloys. The ALTBC function expresses the probability of having a bond of length r_1 almost aligned with another bond of length r_2 with angular deviations smaller than 30°, providing a measure for the degrees of local distortion in the amorphous structures[S13]. Results for AIMD, GAP-MD, and ACE-MD structures are shown in black, blue, and red, respectively. All data shown in this figure present mean values over 6,000 snapshots of AIMD, GAP-MD, and ACE-MD simulations (which were collected from the last 40 ps trajectories of 3 independent melt-quench runs). The error bars in panel (c–d) indicate standard deviations over these 6,000 snapshots. This figure is drawn in a style similar to Ref. [S1], and all structural analyses were performed using the same type of benchmarks as in that prior work: all AIMD and GAP-MD results were produced based on the atomic trajectories as reported in Ref. [S1], and the AIMD and GAP-MD data in plotted panels (a) and (c) are taken from Ref. [S1].

Supplementary References

S1. Zhou, Y., Zhang, W., Ma, E. & Deringer, V. L. Device-scale atomistic modelling of phase-change memory materials. *Nat. Electron.* **6**, 746–754 (2023).

S2. Perdew, J. P., Burke, K. & Ernzerhof, M. Generalized gradient approximation made simple. *Phys. Rev. Lett.* **77**, 3865–3868 (1996).

S3. Pickard, C. J. & Needs, R. J. High-pressure phases of silane. *Phys. Rev. Lett.* **97**, 045504 (2006).

S4. Pickard, C. J. & Needs, R. J. Ab initio random structure searching. *J. Phys.: Condens. Matter* **23**, 053201 (2011).

S5. Deringer, V. L., Pickard, C. J. & Csányi, G. Data-driven learning of total and local energies in elemental boron. *Phys. Rev. Lett.* **120**, 156001 (2018).

S6. Bernstein, N., Csányi, G. & Deringer, V. L. De novo exploration and self-guided learning of potential-energy surfaces. *npj Comput. Mater.* **5**, 99 (2019).

S7. Morrow, J. D., Gardner, J. L. A. & Deringer, V. L. How to validate machine-learned interatomic potentials. *J. Chem. Phys.* **158**, 121501 (2023).

S8. Xu, Y. *et al.* Unraveling crystallization mechanisms and electronic structure of phase-change materials by large-scale ab initio simulations. *Adv. Mater.* **34**, 2109139 (2022).

S9. Beckett, G. *et al. ARCHER2 Service Description*. https://zenodo.org/records/14507040, DOI: 10.5281/zenodo.14507040 (2024).

S10. Lysogorskiy, Y., Bochkarev, A., Mrovec, M. & Drautz, R. Active learning strategies for atomic cluster expansion models. *Phys. Rev. Mater.* **7**, 043801 (2023).

S11. Nicholas, T. C. *et al.* The structure and topology of an amorphous metal-organic framework. Preprint at arXiv:2503.24367 (2025).

S12. Caravati, S., Bernasconi, M., Kühne, T. D., Krack, M. & Parrinello, M. Coexistence of tetrahedral- and octahedral-like sites in amorphous phase change materials. *Appl. Phys. Lett.* **91**, 171906 (2007).

S13. Raty, J. Y. *et al.* Aging mechanisms in amorphous phase-change materials. *Nat. Commun.* **6**, 7467 (2015).
